# ﻿*Kengiochloa*, a new bamboo genus to accommodate the morphologically unique species, *Pseudosasapubiflora* (Poaceae)

**DOI:** 10.3897/phytokeys.221.98920

**Published:** 2023-03-13

**Authors:** Yi-Hua Tong, Zheng-Yang Niu, Zhuo-Yu Cai, Jing-Bo Ni, Nian-He Xia

**Affiliations:** 1 Key Laboratory of Plant Resources Conservation and Sustainable Utilization & Guangdong Provincial Key Laboratory of Applied Botany, South China Botanical Garden, Chinese Academy of Sciences, CN-510650, Guangzhou, China South China Botanical Garden, Chinese Academy of Sciences Guangzhou China; 2 South China National Botanical Garden, CN-510650, Guangzhou, China South China National Botanical Garden Guangzhou China; 3 University of Chinese Academy of Sciences, CN-100049, Beijing, China University of Chinese Academy of Sciences Beijing China

**Keywords:** morphology, phylogeny, plastome, synonyms, taxonomy

## Abstract

*Pseudosasa* was confirmed as polyphyletic by recent phylogenetic analyses, with Chinese species of *Pseudosasa* distantly related to those from Japan. Among the Chinese species of *Pseudosasa*, *Pseudosasapubiflora* is a morphologically unique as well as taxonomically problematic species endemic to South China, of which the generic designation is still uncertain. Molecular analyses based on both plastid and nuclear genomic data demonstrated that this species is closest to the recently published genus *Sinosasa*. Morphologically, the two are somewhat similar to each other in flowering branches developing at the nodes of every order of branches, raceme-like units of inflorescence with 3–5 short spikelets, each spikelet with few florets including a rudimentary one at the apex, and each floret with 3 stamens and 2 stigmas. However, *P.pubiflora* is very different from *Sinosasa* species in many reproductive and vegetative characters, such as the morphology of paracladia (lateral spikelet “pedicels”), the absence or existence of pulvinus at the base of paracladia, the relative length of the upper glume and the lowest lemma, the shape of lodicules and primary culm buds, the branch complement, the morphology of nodes, culm leaves and dried foliage leaf blades, and the number of foliage leaves per ultimate branchlet. The morphological and molecular evidence warrants recognition of a new genus to accommodate this unique species, which is here named *Kengiochloa*. After consulting related literature and examination of herbarium specimens or specimen photos, a taxonomic revision of *K.pubiflora* and its synonyms was made, and it was confirmed that four names, viz. *P.gracilis*, *Yushanialanshanensis*, *Arundinariatenuivagina* and *P.parilis*, should be merged with *K.pubiflora*, while *Indocalamuspallidiflorus* and *Acidosasapaucifolia* are distinct species.

## ﻿Introduction

**Figure 1.**
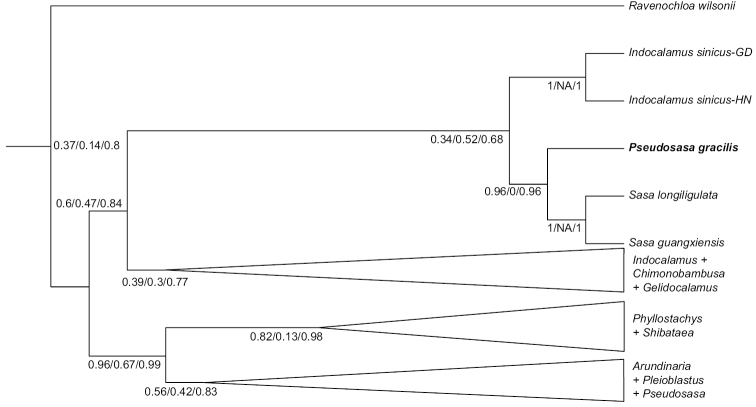
Phylogenetic position of *Pseudosasagracilis*, adapted from Guo et al. 2021.

*Pseudosasa* Makino ex Nakai, with ca. 20 species mainly distributed in East Asia and Vietnam, belongs to the subtribe Arundinariinae of the tribe Arundinarieae (Poaceae: Bambusoideae) (Zhang et al. 2020; Soreng et al. 2022; POWO 2022). Many molecular phylogenetic studies have shown that this genus is polyphyletic, as the Japanese species including the generic type, *P.japonica* (Siebold & Zucc. ex Steud.) Makino ex Nakai, and those from China are always distributed in different clades in the phylogenetic trees (Triplett and Clark 2010; Zhang et al. 2012; Guo et al. 2021; Triplett and Clark 2021). Morphologically, the two groups of species are also different: *P.japonica* has persistent culm leaf sheaths, a solitary branch at each node, or sometimes three branches at the top nodes of the culm, and three to five stamens (which also suggest that *P.japonica* has a hybrid origin between Japanese *Pleioblastus* Nakai and *Sasa* Makino & Shibata or *Sasamorpha* Nakai, as indicated by Triplett and Clark (2010, 2021)), while Chinese *Pseudosasa* species usually have deciduous culm leaf sheaths, one to three branches per node, and three stamens (Chen et al. 1996; Zhu et al. 2006; Zhang et al. 2012; Kobayashi 2017). Among those sampled *Pseudosasa* species, one species endemic to South China, viz. *P.gracilis* S. L. Chen & G. Y. Sheng, drew the attention of many researchers. This species was consistently associated with some Chinese *Sasa* species (which were recently transferred to the newly established genus, *Sinosasa* L. C. Chia ex N. H. Xia et al., see Qin et al. 2021), and far related with other *Pseudosasa* species in previous studies (Zhang et al. 2012; Guo et al. 2021) (Fig. 1). Zhang et al. (2012) argued that *P.gracilis* was not a typical member of *Pseudosasa* in terms of morphology, and suggested that more work was required to clarify the ascription of this species and its phylogenetic placement. In Flora of China, Zhu et al. (2006) considered that *P.gracilis* was possibly a synonym of *P.pubiflora* (Keng) Keng f. ex D. Z. Li & L. M. Gao, which they thought may not belong to *Pseudosasa* but *Indocalamus* Nakai.

*Pseudosasapubiflora* has a relatively complicated taxonomic history. In 1936, Yi-Li Keng, the first Chinese botanist who studied the bamboo classification in China, described *Arundinariapubiflora* Keng from northern Guangdong based on only one collection (*To & Tsang 12284*) with flowers (Keng 1936). His son, Pai-Chieh Keng then transferred it to *Pseudosasa* Makino ex Nakai without giving any reason (Keng 1957 & 1959). However, the new combination was invalid due to lack of a clear and direct reference to the basionym (Zhu et al. 2006). Yang and Chao (1994) recognized this species in *Arundinaria* Michx.., and considered several other names, including *P.gracilis*, *Indocalamuspallidiflorus* McClure, *Yushanialanshanensis* T. H. Wen and *Acidosasapaucifolia* W. T. Lin as well as several combinations based on these names, as synonyms of this species.

The *P.pubiflora* published in Flora of China (Zhu et al. 2006) was a combination largely based on Yang and Chao’s (1994) treatment, except that *P.gracilis* was suggested as “possibly a synonym of *P.pubiflora*”. Besides, two more names, viz. *Arundinariateuivagina* W. T. Lin and *P.parilis* T. P. Yi & D. H. Hu, were further synonymized with *P.pubiflora*. Consequently, the distribution of this species was extended to include three provinces, i.e., Guangdong, Hunan and Jiangxi. Moreover, Zhu et al. (2006) argued that, for *P.pubiflora*, “the woolly internode apex is unusual in *Pseudosasa* and rather suggestive of *Indocalamus* Nakai”. Thus, the relationships between *P.pubiflora* and its synonyms and the generic designation of this species need a further study.

## ﻿Materials and methods

Field trips to the type locality of *P.pubiflora* as well as its several synonyms including *I.pallidiflorus*, *Arundinariateuivagina* and *P.parilis* were conducted from 2018 to 2022. The complete specimens including rhizomes, culms with culm leaves, branches and foliage leaves as well as fresh young foliage leaves used for molecular analyses were collected. The types and other specimens of the pertinent species housed at CANT, IBSC, N, SYS and ZJFI, as well as photographs of specimens housed at K, US and W, were examined. Herbaria acronyms follow Thiers (2022). The morphological description is based on specimens and referred to related literature (Keng 1936; Chen et al. 1983; Wen 1985, 1986; Lin and Wu 1990; Yi 1995; Chen et al. 1996; Zhu et al. 2006). General morphological terms follow Beentje (2016). The terms applied to the flowering structure in this study mainly follow the synflorescence concept applied to grasses (Vegetti and Anton 1996; Tivano et al. 2009; Cai and Xia 2021).

To study the phylogenetic position of *P.pubiflora* within the tribe Arundinarieae, the whole chloroplast genomes were used for building the phylogenetic tree. Five *Pseudosasa* species including one from Japan, also the type species of the genus, viz. *P.japonica*, and four from China were sampled. In total, there were 29 species represented by 30 samples of all the five subtribes in the tribe Arundinarieae (two samples for *P.pubiflora*) and one sample of *Bambusabambos* (L.) Voss from the tribe Bambuseae was the outgroup. Species names, voucher information and GenBank accession numbers are provided in Table 1.

**Table 1.** Species names, voucher information and GenBank accession numbers of the 30 whole chloroplast genomes used in this study.

Taxon	Voucher information	GenBank accession
**Ingroup**		
*Acidosasaglauca* B. M. Yang	CZY56 (IBSC)	*OP850353
*Ampelocalamusactinotrichus* (Merr. & Chun) S. L. Chen T. H. Wen & G. Y. Sheng	MPF10003 (KUN)	MF066245
*Chimonobambusaquadrangularis* (Franceschi) Makino	Not provided by the author	MW928533
*Ferrocalamusrimosivaginus* T. H. Wen	Zhang08019 (KUN)	HQ337794
*Ferrocalamusstrictus* Hsueh & Keng f.	NH001 (IBSC)	*OP850355
*Gaoligongshaniamegalothyrsa* (Hand.-Mazz.) D. Z. Li, Hsueh & N. H. Xia	MPF10056 (KUN)	JX513419
*Gelidocalamusstellatus* T. H. Wen	BH102 (IBSC)	*OP850347
*Gelidocalamustessellatus* T. H. Wen & C. C. Chang	MPF10049 (KUN)	JX513420
*Hsuehochloacalcarea* (C. D. Chu & C. S. Chao) D. Z. Li & Y. X. Zhang	MPF10050 (KUN)	KJ496369
*Indocalamuslongiauritus* Hand.-Mazz.	MPF10168 (KUN)	HQ337795
*Indocalamuslatifolius* (Keng) McClure	CZY76 (IBSC)	*OP850354
*Indocalamussinicus* (Hance) Nakai	ZMY037 (KUN)	MF066250
*Indosasacrassiflora* McClure	BH58 (IBSC)	*OK558536
Phyllostachysnidulariaf.farcta H. R. Zhao & A. T. Liu	2020-JZ01 (NF)	LC590826
*Pleioblastuschino* (Franch. & Sav.) Makino	NH029 (IBSC)	*OP850356
*Pseudosasaamabilis* (McClure) Keng f.	NH032 (IBSC)	*OP850358
*Pseudosasacantorii* (Munro) Keng f.	MPF10006 (KUN)	MF066255
*Pseudosasajaponica* (Siebold & Zucc. ex Steud.) Makino ex Nakai	Pjc-1 (ZJFC)	KT428377
*Pseudosasananunica* (McClure) Z. P. Wang & G. H. Ye	XNH36 (IBSC)	*OP850361
*Pseudosasapubiflora* (Keng) Keng f. ex D. Z. Li & L. M. Gao	CZY146 (IBSC)	*OP850350
*Pseudosasapubiflora* (Keng) Keng f. ex D. Z. Li & L. M. Gao	XNH63 (IBSC)	*OP850362
*Shibataeachinensis* Nakai	NH036 (IBSC)	*OP850359
*Sinobambusatootsik* (Makino) Makino ex Nakai	NH031 (IBSC)	*OP850357
*Sinosasafanjingshanensis* N. H. Xia, Q. M. Qin & J. B. Ni	BH124 (IBSC)	*OP850348
*Sinosasa* sp.	CZY173 (IBSC)	*OP850352
*Sinosasalongiligulata* (McClure) N. H. Xia, Q. M. Qin & J. B. Ni	CZY163 (IBSC)	*OP850351
*Ravenochloawilsonii* (Rendle) D. Z. Li & Y. X. Zhang	MPF10146 (KUN)	JX513421
*Oligostachyumsulcatum* Z. P. Wang & G. H. Ye	CZY113 (IBSC)	*OP850349
*Yushaniabasihirsuta* (McClure) Z. P. Wang & G. H. Ye	XNH144 (IBSC)	*OP850360
**Outgroup**		
*Bambusabambos* (L.) Voss	B1-1 (ISC)	KJ870988

Note: Asterisk (*) indicates the DNA data obtained in this study.

Total genomic DNA was isolated from silica gel-dried leaves using TIANGEN Genomic DNA Extraction Kit (TIANGEN, Beijing, China). The extracted genomic DNA was fragmented randomly by Covaris M220 (Covaris, Woburn, MA), and the fragments with insert size of 350 bp were selected by using AxyPrep Mag PCR Clean Up Kit. The selected fragments were enriched by PCR after undergoing end repair, the addition of polyA tail and adaptor ligation. The paired-end (2 × 150 bp) libraries were constructed on NovaSeq 6000 platform. Finally, 20 Gb genome skimming data were generated for each sample.

After quality control of 20 Gb clean data by Trimmomatic v 0.39 (Bolger et al. 2014), the whole chloroplast (cp) genomes were assembled using the software GetOrganelle v 1.7.4 pipeline (Jin et al. 2018), with the reference cp genome sequence of *Ampelocalamussinovietnamensis* (MW525255) and K-mer sizes of 45, 65, 85, 105 and 125. The generated whole cp genome was annotated using the program Plastid Genome Annotator (Qu et al. 2019) based on the annotation of *A.sinovietnamensis*. The transferred RNAs (tRNAs) were adjusted using the software tRNAscan-SE service (Schattner et al. 2005). The initiation and termination codons of all the coding genes were manually verified in Geneious Prime v 9.1.4 (Kearse et al. 2012).

All the cp genomes were aligned using MAFFT v. 7.450 (Katoh and Standley 2013). Maximum Likelihood (ML) analysis was conducted by RAxML v 8.2.12 (Stamatakis 2014) with the rapid bootstrap algorithm. GTRGAMMAI was selected as the best-fit model recommended by jModelTest v2.1.6 (Darriba et al. 2012). The number of 12345 was specified as the random seed of parsimony tree inference with 1000 replicates performed. For Bayesian Inference (BI), the data matrix was calculated using MrBayes v3.2.2 software (Ronquist et al. 2012). The best-fit model was selected as SYM+G under the Akaike information criterion (AIC) using MrModeltest v 2.3 (Nylander 2004). Rates of variations across sites were trimmed as gamma. For each analysis, two simultaneous runs of four Monte Carlo Markov Chains (three heated and one cold) were run for 20 million generations with a random tree as the starting point and saving trees every 1000 generations. After discarding the first 25% samples as burn-ins, the optimized topology was generated. The final results were visualized with Figtree 1.4.3 (http://tree.bio.ed.ac.uk/software/figtree/).

## ﻿Results

The chloroplast genomes ranged from 139,093 bp (*Pleioblastuschino* Makino) to 140,064 bp (*Gaoligongshaniamegalothyrsa* (Hand.-Mazz.) D. Z. Li, Hsueh & N. H. Xia) with an alignment length of 144,771 bp. The plastid data matrix was characterized by sequence divergence with 9,751 variable sites (6.74%), including 6,477 parsimony informative sites (4.47%) and 3,274 singleton variable sites (2.27%).

**Figure 2.**
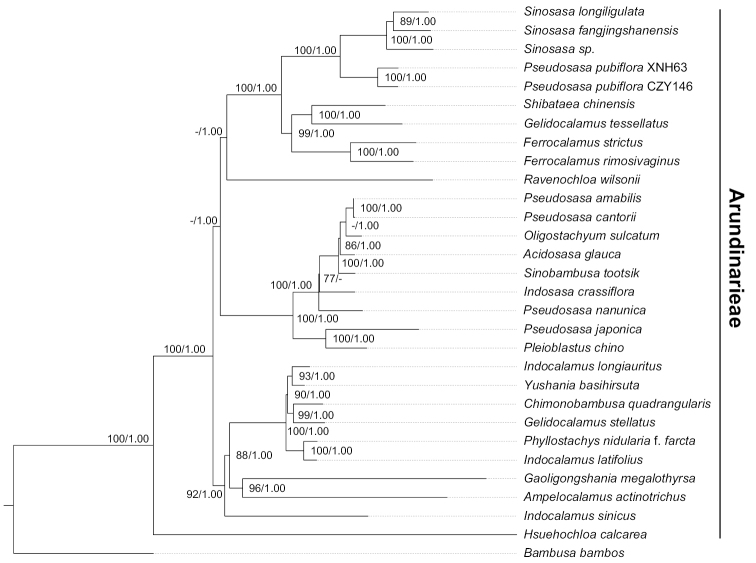
Phylogeny of Arundinarieae based on whole chloroplast genome derived from maximum likelihood and Bayesian analyses. Only bootstrap values (BS) ≥ 70% and posterior probabilities (PP) ≥ 0.95 are indicated at each node.

The reconstructed phylogenetic tree is shown in Fig. 2. Similar to some previous analyses on plastid sequence data (Triplett and Clark 2010; Zhang et al. 2012), *Pseudosasa* was resolved as polyphyletic in this analysis. *Pseudosasapubiflora* was sister to the clade of *Sinosasa* with strong support (maximum likelihood bootstrap value/Bayesian inference posterior probability BS/PP = 100%/1.00). The other *Pseudosasa* taxa form a clade with five other genera of the subtribe Arundinariinae, namely *Oligostachyum* Z. P. Wang & G. H. Ye, *Acidosasa* B. M. Yang, *Sinobambusa* Makino, *Indosasa* Maclure and *Pleioblastus* (MLBS/PP = 100%/1.00).

## ﻿Discussion

### ﻿Taxonomic revision of *Pseudosasapubiflora* and its synonyms

**Figure 3.**
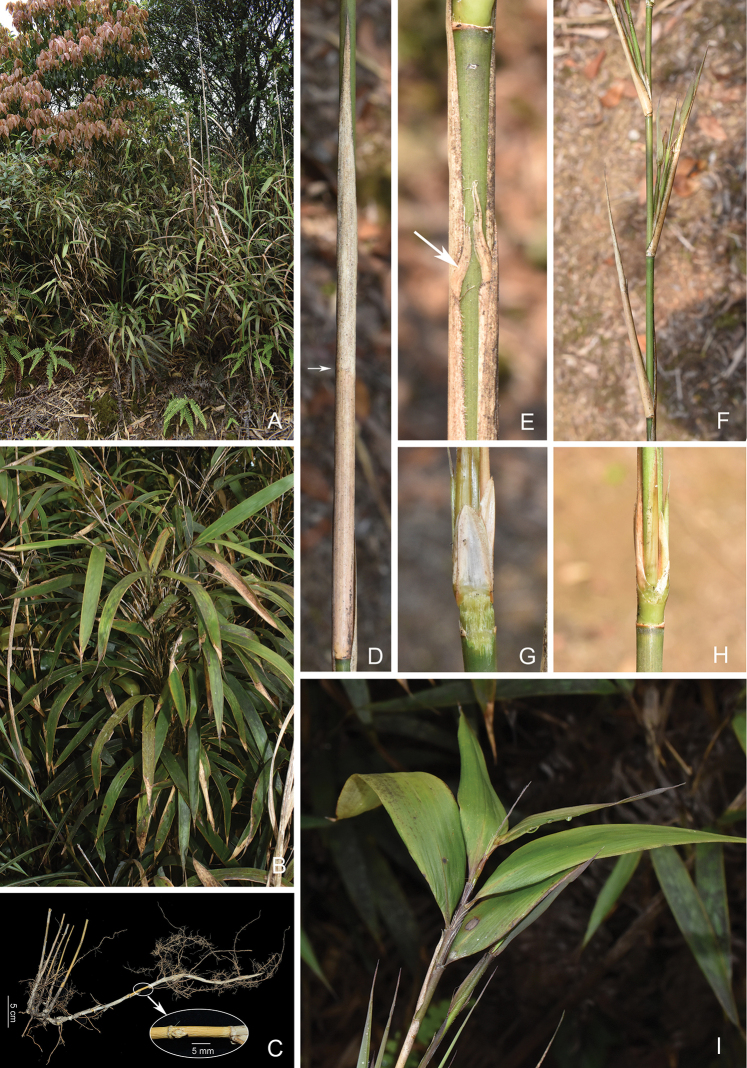
*Kengiochloapubiflora***A** habitat **B** habit **C** leptomorph rhizome and pluricaespitose culms, the arrow showing an internode with a bud **D** culm leaf, the arrow showing the junction of sheath and blade **E** culm leaf oral setae **F** intravaginal branching **G** prophyll of primary branch **H** three young branches at a node **I** foliage leaf branch. Photos by Y. H. Tong.

**Figure 4.**
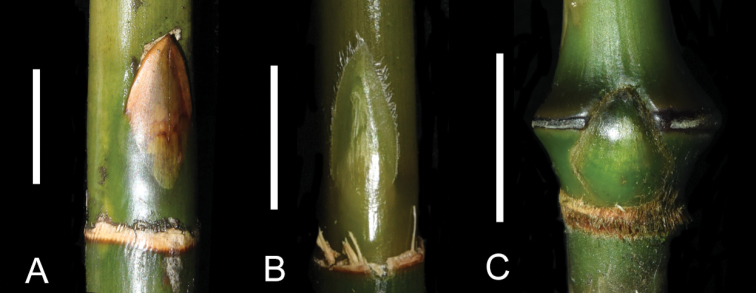
Culm buds of three bamboos **A***Indocalamussinicus***B***Kengiochloapubiflora***C***Sinosasa* sp. Photo **A** by Xi-Rong Zheng, **B, C** by Zhuo-Yu Cai. Scale bars: 1 cm (**A, C**); 5 mm (**B**).

After consulting related literature and examining the types and other herbarium specimens or specimen photographs, we agree with Yang and Chao (1994) that *P.gracilis*, *Yushanialanshanensis* should be merged into *P.pubiflora*, and with Zhu et al. (2006) that *Arundinariateuivagina* and *P.parilis* are also synonyms of *P.pubiflora*, since all these species share the same key diagnostic characters, mainly including the leptomorph rhizome, the pluricaespitose, short (< 2 m) and thin (< 0.8 mm) culm (Fig. 3C), the flat node, the narrowly ovate culm buds (Fig. 4B), the branch complement with two to four branches at each mid-culm node, the persistent papery culm leaf sheath with fragile oral setae that are adnate at base (Fig. 3E), the erect and amplexicaul culm leaf blades that are usually longer than sheath (Fig. 3D), and the ultimate branchlet with one to four lanceolate or narrowly lanceolate foliage leaves (Fig. 3B). Among these characters, its oral setae and culm leaf blade are very special in Arundinarieae.

However, we do not agree that *I.pallidiflorus* and *Acidosasapaucifolia* are identical to *P.pubiflora*. *Indocalamuspallidiflorus* (≡ *Pseudosasapallidiflora* (McClure) S. L. Chen & G. Y. Sheng) markedly differs from *P.pubiflora* in the woolly (vs. glabrous) infranodal region, the culm leaf sheath oral setae present (vs. absent) and the culm leaf blade much shorter (vs. usually longer) than sheath. Actually, the description of *P.pubiflora* in Flora of China is mainly based on that of *I.pallidiflorus* made by McClure (1940), which conflicts a lot with the protologue of *P.pubiflora* (Keng 1936). We cannot find the type specimens of *Acidosasapaucifolia* despite an exhaustive search in CANT where they should be preserved, as indicated by the author (Lin 1992). According to the description and illustration in the protologue (Lin 1992), this species significantly differs from *P.pubiflora* by the deciduous culm leaf with abaxially more or less hispid (vs. usually glabrous) sheaths and much longer ligules (2 mm vs less than 0.5 mm) and without (vs. with) oral setae, the absence (vs. existence) of foliage leaf oral setae and much longer foliage leaf inner ligules (3 mm vs. 0.3–1.5 mm). Thus, this species should not be synonymized with *P.pubiflora* either. The identities of these two taxa need further study.

### ﻿Generic designation of *Pseudosasapubiflora*

Recent research has proved that the formerly recognized *Arundinaria* in a broad sense should not be adopted any more, which at present is thought to be a small genus with only three species restricted to North America (Zhang et al. 2012, 2016, 2020; Guo et al. 2021; Soreng et al. 2022). Thus, *Pseudosasapubiflora* should not be placed into *Arundinaria*. This species has many characteristics that are very unusual for *Pseudosasa*, such as the lanceolate (vs. narrowly terete or linear in other *Pseudosasa* species, the same below) and rather short (1.6–2 cm long vs. usually much longer and up to 20 cm) spikelets with robust and erect (vs. slender and porrect) pedicels and very few florets (2–4 vs. 3–30), each floret with two (vs. three) stigmas, the papery (vs. leathery) culm leaf sheath and the glabrous and nonpowdery (vs. conspicuously powdery) infranodal regions (Table 2). The phylogenetic analyses of both our study based on the chloroplast genomes and Guo et al. (2021) based on nuclear markers from ddRAD data demonstrated that this species had a distant relationship with other *Pseudosasa*. Thus, *P.pubiflora* should not be a member of *Pseudosasa*.

**Table 2.** Comparison of key morphological characters of *Pseudosasapubiflora*, *Sinosasa*, *Indocalamussinicus* and *Pseudosasa*. The characters unique to *P.pubiflora* among these taxa are indicated in bold.

	*P.pubiflora*	*Sinosasa*	*Indocalamussinicus*	*Pseudosasa*
Culm bud	**Narrowly ovate**	Trullate	Triangle, lower half adnate to culm	Trullate to narrowly trullate
Branches at mid-culm nodes	2 to 4	Solitary	Solitary	1 to 3
Nodes	Flat	Prominent, supranodal ridge strongly raised	Flat	Flat or weakly prominent
Infranodal region	Glabrous, nonpowdery	With a sericeous or villous band	Glabrous, nonpowdery	Powdery
Culm leaf sheath	Persistent, papery	Persistent, papery	Persistent, leathery-papery	Persistent, usually leathery
Culm leaf oral setae	**Adnate at base**	Distinct	Distinct or absent	Distinct or absent
Culm leaf blades	Erect, amplexicaul, **usually longer than sheath**	Erect or slightly spreading, not amplexicaul, much shorter than sheath	Erect or reflexed, not amplexicaul, narrowly triangular-lanceolate, much shorter than sheath	Erect or reflexed, amplexicaul or not, narrowly triangular to strap-shaped, shorter than or as long as sheath
Number of foliage leaves per ultimate branchlet	1 to 4	5 to 17	7 to 14	2 to 10
Inner ligule	Very short, 0.3–1.5 mm	Long, (8–)10–20 mm	Short, 2–3 mm	Short to long, 1–5 mm
Foliage leaf blades	Flat when dry	Wavy when dry	Flat when dry	Flat when dry
Flowering branch	Developing at the nodes of every order of branches	Developing at the nodes of every order of branches or culm	Terminal on culm or foliage leaf branches	Developing at the nodes of every order of branches or culm
The unit of inflorescence of the synflorescence	Raceme-like with 3–5 spikelets	Raceme-like with 3–5 spikelets	Panicle-like with many spikelets	Raceme or panicle-like with several to many spikelets
Paracladia	**Robust and erect, appressed to the axis**	Slender, porrect to reflexed	Slender, porrect	Slender, porrect
Pulvinus at the base of pracladia	Absent	Present	Present	Absent
Spikelet	Lanceolate, 1.6–2 cm long	Lanceolate, 0.5–1.5 cm long	Lanceolate, 1.5–2.5 cm long	Narrowly terete or linear, 2.5–20 cm long
Number of floret per spikelet	2–4	2–3	3–4	3–30
Terminate rudimentary floret	Exist	Exist	Exist	Exist or not
The relative length of upper glume and the lowest lemma	Upper glume shorter than the lowest lemma	Upper glume nearly equal to the lowest lemma	Upper glume shorter than the lowest lemma	Upper glume shorter than the lowest lemma
Lodicule	Oblong-obovate, spatulate or obovate, apex rounded	Ovate or narrow oblong, apex acute	Ovate to oblong, apex obtuse	Oblong, obovate-lanceolate, spathulate or lanceolate, apex acute or obtuse
Stigma number	2	2	2	3 to 5

Interestingly, both of the two phylogenetic studies suggested a close relationship between *Pseudosasapubiflora* and *Sinosasa*. In the analysis of Guo et al. (2021), *Indocalamussinicus* (Hance) Nakai is closest to the *Pseudosasapubiflora* + *Sinosasa* clade (called “*Pseudosasagracilis* + Sasasubg.Sasa” clade (Fig. 1) in that paper) with a strong support. Morphologically, this small clade is characterized by having persistent papery culm leaf sheath, short (less than 2.5 cm) and lanceolate spikelet with few florets (2–4) including a terminate rudimentary one and each floret with two stigmas in the subtribe Arundinariinae. However, the flowering branch of *Indocalamussinicus* is terminal on culm or foliage leaf branches with a large and panicle-like unit of inflorescence composed of many spikelets, while the flowering branches of the other two develop at the nodes of every order of branches with a raceme-like unit of inflorescence composed of only 3–5 spikelets. The culm buds of *Indocalamussinicus* are also very special; they are triangular with lower half completely adnate to the culm (Fig. 4A).

Last, there are so many different vegetative and productive characters between *Pseudosasapubiflora* and *Sinosasa* that they cannot belong to the same genus. Specifically, *Pseudosasapubiflora* differs from *Sinosasa* in having narrowly ovate culm buds (vs. trullate) (Fig. 4), branch complement with 2–4 branches at mid-culm nodes (vs. solitary), flat nodes (vs. prominent with a strongly raised supranodal ridge), infranodal region being glabrous and nonpowdery (vs. with a sericeous or villous band), culm leaf with oral setae adnate at base (vs. distinct) and amplexicaul blade being usually longer than sheath (vs. not amplexicaul and much shorter than sheath), 1–4 foliage leaves per ultimate branchlet (vs. 5–17) with blades flat when dry (vs. wavy) and much shorter inner ligule (0.3–1.5 mm vs. (8–)10–20 mm), robust and erect (vs. slender and porrect to reflexed) paracladia being appressed to the axis and without (vs. with) pulvinus at base, upper glumes typically shorter than (vs. nearly equal to) the lowest lemma and lodicules with a rounded (vs. acute) apex (Detailed comparison is provided in Table 2). In conclusion, both the morphological and molecular phylogenetic evidence strongly supports the recognition of a new genus to accommodate *Pseudosasapubiflora*.

### ﻿Taxonomic treatment

#### ﻿ Kengiochloa

Taxon classificationPlantaePoalesPoaceae

Y.H.Tong & N.H.Xia gen. nov.

77DCDA38-34FE-5E58-8A4C-57B526FE38DE

urn:lsid:ipni.org:names:77315562-1

##### Type.

*Kengiochloapubiflora* (Keng) Y. H. Tong & N. H. Xia (≡ *Arundinariapubiflora* Keng).

##### Diagnosis.

This new genus is close to *Sinosasa*, but differs in having narrowly ovate culm buds, branch complement with 2–4 branches at mid-culm nodes, flat nodes, glabrous and nonpowdery infranodal region, culm leaf oral setae adnate at base, amplexicaul culm leaf blades that are usually longer than sheath, 1–4 foliage leaves per ultimate branchlet with blades flat when dry and short inner ligule, robust and erect paracladia (lateral spikelet pedicels) appressed to the axis and without pulvinus at base, upper lemma typically shorter than the lowest lemma and lodicules with a rounded apex.

##### Description.

Shrubby bamboo. Rhizomes leptomorph. Culms pluricaespitose, short and thin, less than 2 m tall and 8 mm in diam.; nodes flat. Culm buds narrowly ovate. Branches intravaginal, 2–4 at each mid-culm node. Culm leaf sheath persistent, papery; auricles absent; oral setae fragile, adnate at base; blade erect and amplexicaul, usually longer than sheath; ligule convex, short. Foliage leaves 1–4 per ultimate branchlet; auricles obscure; inner ligule short. Flowering branches developing at the nodes of every order of branches; the unit of inflorescence of synflorescence raceme-like with 3–5 spikelets; paracladia robust and erect, appressed to the axis and without pulvinus at base; florets 2–4 per spikelet including a rudimentary one at the apex; glumes 2, upper one shorter than the lowest lemma; lemma longer than palea; palea 2-keeled; lodicules 3, apex rounded; stamens 3, anthers pale yellow; styles 2, base slightly connate; stigmas 2, plumose. Caryopsis unknown.

##### Etymology.

*Kengiochloa* is named in honor of Professor Yi-Li Keng (1897–1975), a renowned botanist and the first Chinese who studied the bamboo taxonomy in China. The type species of this genus was first described by him, too. Its Chinese name is given as 以礼竹属 (*pinyin*: yĭ lĭ zhú shŭ).

#### ﻿ Kengiochloa pubiflora

Taxon classificationPlantaePoalesPoaceae

(Keng) Y.H.Tong & N.H.Xia comb. nov.

1EF9A752-94E6-5121-9072-9757518A9C19

urn:lsid:ipni.org:names:77315564-1

≡ Arundinaria pubiflora Keng, Sinensia 7: 414, fig. 4. 1936; Yang & Chao, J. Bamb. Res. 13(1): 5. 1994, excl. syn. of Indocalamuspallidiflorus. ≡ Pseudosasapubiflora (Keng) Keng f. ex D. Z. Li & L. M. Gao in Zhu et al., Fl. China 22: 118. 2006, excl. descr. and syn. of Indocalamuspallidiflorus and Acidosasapaucifolia; Keng f. in Keng, Clav. Gen. Sp. Gram. Prim. Sin. 154. 1957 & Fl. Ill. Pl. Prim. Sin. 32 & pl. 22. 1959, *nom. inval.* Type: China, Guangdong, on top of the hill at the rear of Iu [Yao] village, Lung Tau Mt. [Longtou Mountain], 30 May 1924, *To & Tsang 12284* (holotype US00130296 (sheet 1) & US00065420 (sheet 2), image!, isotypes US0031360, image!, US00144993, image!, W19390008723, image!, SYS!). Figs 3, 4B; also see fig. 150 in Vol. 22 of Flora of China Illustrations (available at http://www.efloras.org/object_page.aspx?object_id=95963&flora_id=2).
= Pseudosasa gracilis S. L. Chen & G. Y. Sheng, Acta Phytotax. Sin. 21: 405. 1983; Chen et al., Fl. Reipubl. Popularis Sin. 9(1): 658. 1996; Zhu et al., Fl. China 22: 119. 2006. Type: China, Hunan, Yizhang County, Zeping, 6 May 1977, *Z. P. Wang*, *H. R. Zhao & G. Y. Sheng 77004* (holotype NAS, not seen, isotypes N019022078!, N019022079!, N019022080!).
= Yushania lanshanensis T. H. Wen, J. Bamboo Res. 4(2): 13. 1985. ≡ Arundinarialanshanensis (T. H. Wen) T. H. Wen, J. Bamboo Res. 5(1): 19. 1986. Type: China, Hunan, Lanshan County, Ziliang Township, 1125 m, 17 June 1984, *S. Q. Chen CX84860* (holotype ZJFI!, isotypes ZJFI!).
= Arundinaria tenuivagina W. T. Lin & Z. M. Wu, J. S. China Agric. Univ. 11(3): 48. 1990. Type: China, Guangdong Province, Xinyi City, Dawuling, 1400 m, 12 April 1987, *Z. M. Wu 0167* (holotype CANT00002410!, isotypes CANT00002408!, CANT00002409!, CANT00002411!, CANT00002412!, CANT00002413!, CANT00002414!, CANT00002415!).
= Pseudosasa parilis T. P. Yi & D. H. Hu in Yi, J. Bamboo Res. 14(1): 20. 1995. Type: China, Jiangxi, Suichuan County, Dabali, 1400 m, 21 November 1991, *Q. Hu & D. H. Hu 032* (holotype SAUT, not seen, isotype Herbarium of Ji’an Forestry Institution, not seen).

##### Description.

Rhizomes amphipodial, internodes terete, 2–2.5 cm long, ca. 2.5 mm in diam. Culms 1.2–2 m tall, up to 8 mm in diam.; internodes terete, 8–24 cm long, green, not powdery, glabrous; wall thick, cavity with woolly or irregularly lamellate pith; supranodal ridge flat, remains of sheath base persistent, sheath scar pubescent or glabrous. Culm buds narrowly ovate. Branches intravaginal, 2–4 at each mid-culm node, base attached to culm. Culm leaf sheaths gradually deciduous or rather persistent, 1/2 to 4/7 as long as internodes, thickly papery, glabrous or sometimes sparsely brown appressed hispidulous, margins ciliolate; auricles absent; oral setae 5–11, lateral 1–4 curved and downward, others straight forward, 6–9 mm long, fragile, base usually adnate; ligule short, less than 0.5 mm, unevenly laciniate, glabrous; blade erect, amplexicaul, striate, broadly ovate-lanceolate, usually longer than sheath, both surfaces glabrous, margins ciliolate, apex acuminate. Foliage leaves 1 to 4 per ultimate branch; sheath 2–4 cm long, upper half more or less white-pilose abaxially, margins ciliolate, glabrescent; outer ligule ca. 0.5 mm long, margin white-pilose; auricles obscure; oral setae present, to 1.4 cm long; inner ligule short, 0.3–1.5 mm; pseudopetiole 1.5–3 mm long, slightly pilose adaxially, glabrous abaxially; blade lanceolate or narrowly lanceolate, 8–24 × 0.9–2 cm, slightly pilose at base adaxially, hispidulous abaxially, glabrescent, base cuneate, margins serrulate, apex acuminate; lateral veins 4–5 pairs, veinlets conspicuous. The unit of inflorescence of the synflorescence 3–9 cm long; main axis glabrous, flattened on the branching side, basal internode 0.8–1.8 cm long. Paracladia 2–4, 0.4–1 cm long, robust, close to the axis, puberulent or appressed-pubescent at the apex, base without a pulvinus. Spikelets 1.6–2 cm long, stramineous; developed (fertile) florets 1–3, uppermost one rudimentary; rachilla segments thickened upwards, 3–4 mm long, glabrous except the upper part white-tomentose; glumes 2, the lower one lanceolate to narrowly lanceolate, 7–10 mm long, glabrous at lower part, pubescent towards the apex, apex acuminate; the upper one with the same shape and indumentum as the lower one, 7–12 mm long; callus bearded with white- or grey-tomentose hairs; lemmas ovate, the lowest one 10–12 mm long, densely adpressed-pubescent abaxially, 9-veined, apex acuminate-mucronate; palea 7–8 mm long, apex obtuse, glabrous except the ciliate and strongly curved keels; lodicules 3, ca. 2 mm long, anterior 2 oblong-obovate, posterior 1 spatulate or obovate, rounded at apex, lower part brown-nerved, upper part hyaline and minutely ciliate; anthers 3, pale yellow, 5 mm long; ripe ovary 2 mm long, brownish when dry; styles 2, persistent, ca. 1 mm long, base slightly connate, stigmas very thin, plumose, ca. 3 mm long. Fruit unknown. Description of inflorescence follows Keng (1936).

##### Phenology.

Culm shoots produced in April to July, flowering in May.

##### Distribution and habitat.

This species is distributed in Hunan, Jiangxi and Guangdong, China. It grows under evergreen broadleaved forests at an elevation from 1100–1600 m.

##### Chinese name.

以礼竹 (pinyin: yĭ lĭ zhú).

##### Additional specimens examined.

*Kengiochloapubiflora*: China. Guangdong: Ruyuan County, Tianjingshan, 4 May 1978, *Z. P. Wang & S. T. Liu 780047* (N019022073, N019022074, N019022075, N019022076); Shixing County, Longtou Moutain, elev. 1131 m, 25 April 2021, *Z. Y. Cai & X. H. Ye czy-146* (IBSC); Xinyi City, Mianbeiding, 20 April 2020, *N. H. Xia*, *Y. H. Tong*, *J. B. Ni*, *S. J. Zeng & B. M. Wang XNH-63* (IBSC). Hunan: Yizhang County, Mang Mountain, elev. 1600 m, 4 July 1964, *B. M. Yang 026501* (N019022077). Jiangxi: Suichuan County, Dabali, 22 April 2022, *C. Long BH-148* (IBSC); ibid. 13 November 2022, *C. Long s.n.* (IBSC). *Indocalamuspallidiflorus*: CHINA. Guangdong: Longmen County, Nankun Mountain, 12 April 1932, *W. T. Tsang 20216* (SYS00095376, US00065467 (image), US00312706 (image), US00312707 (image)); ibid., 18 April 2018, *X. R. Zheng*, *J. B. Ni & Y. Y. Zhang zxr-145* (IBSC). *Indocalamussinicus*: CHINA. Hongkong: hill sides, *C. Wright s.n.* (K00092161 (image)); without precise locality, *Hance s.n.* (K00092162 (image), K00092163 (image)).

## Supplementary Material

XML Treatment for Kengiochloa

XML Treatment for Kengiochloa pubiflora
